# Maternal and neonatal consequences of cystocele and rectocele in the delivery process

**DOI:** 10.1097/MD.0000000000036720

**Published:** 2023-12-22

**Authors:** Yusuf Başkiran, Kazim Uçkan, İzzet Çeleğen, Fatma Başak Tanoğlu

**Affiliations:** a Yuzuncu Yil University, Faculty of Medicine, Gynecology and Obstetrics Clinic Van, Van, Turkey; b Yuzuncu Yil University, Faculty of Medicine, Public Health Van, Van, Turkey; c Çaldiran State Hospital, Van, Turkey.

**Keywords:** cystocele, neonatal outcomes, rectocele, stages of birth, vaginal delivery

## Abstract

The study aimed to investigate the effects of cystocele and rectocele on the stages of vaginal birth and maternal and newborn outcomes. A total of 672 multiparous pregnant women between the ages of 18 to 40 who underwent normal vaginal delivery in our tertiary center between November 2022 and February 2023, were included in this prospective study. Among the participants, 348 (51.8%) had no abnormalities, 78 (11.6%) had rectocele only, 112 (16.7%) had cystocele only, and 134 (19.9) had both cystocele and rectocele. Patients with the coexistence of cystocele and rectocele experienced a notably extended duration for both the first stage and second stage of labor, although the extension in the second stage was not statistically significant. Among the maternal complications, the development of maternal laceration and chorioamnionitis was significantly more common in the patient group with cystocele and rectocele compared to the other groups. When the groups were assessed for postpartum bleeding, while the bleeding risk increased from the normal group to the rectocele + cystocele group, this increase was not statistically significant. There was no difference between the groups in terms of neonatal outcomes. The delivery time of pregnant women with cystocele and rectocele, in the absence of additional risk factors, was determined to be significantly longer than that of the control group. We think that these patients should receive more vigilant monitoring, and this criterion should be kept in mind when assessing the indication for a cesarean section.

## 1. Introduction

Vaginal birth is an evolutionary process that has been experienced for thousands of years to establish a sustainable and safe method of human reproduction. It has a history of over 200,000 years as a safe and effective birth method for more than 7.6 billion people worldwide.^[1]^

As pregnancy progresses in women, the uterine volume gradually increases. Chronic pressure on the pelvic floor system, coupled with the effect of gravity, damages healthy soft tissues. At the same time, many changes in hormone levels make this even more pronounced. The function of collagen changes in the pelvic floor system. Besides the abnormal function of the supporting structures in the pelvic floor system, there may be an increased risk of pelvic organ system prolapse. Prolapse and stress incontinence are complications closely related to vaginal delivery.^[2]^

Pelvic organ prolapse (POP) is the dip of pelvic structures into the vagina due to ligament or muscle weakness. POP is subcategorized concerning the landing compartment. Cystocele characterizes anterior wall herniation, rectocele means posterior vaginal wall descent and vaginal vault prolapse characterizes descent of the apex of the uterus, cervix, or vagina. They can appear alone or in combination. Though the etiology of POP is multifactorial, there is a high relationship between pregnancy and vaginal delivery, which can directly lead to pelvic floor muscle and connective tissue injury.^[3,4]^

Furthermore, previous pelvic surgeries or conditions related to episodes of ever-increasing intra-abdominal pressure, such as heavy lifting, obesity, chronic cough, and constipation may increase the risk of developing POP. Most patients presenting with prolapse are asymptomatic. However, the symptoms become more annoying as the swelling protrudes beyond the vaginal opening. The first evaluation includes a detailed history and a systematic pelvic examination. An evaluation of complications associated with POP, including urinary incontinence, bladder exit obstruction, and fecal incontinence, should be made.^[3,4]^

Vaginal birth carries a high risk of pelvic floor weakness. It has been demonstrated that the weakening of the pelvic floor muscles increases with parity.

Ingrid et al showed that the frequency of pelvic floor disorders increased by 12.8%, 18.4%, 24.6%, and 32.4% in 0, 1, 2, and 3 births, respectively.^[5]^

There are 4 degrees of cystocele:

Stage 0: Absence of prolapse.

Stage 1: The most distal part of the prolapse is more than 1 cm above the hymen level.

Stage 2: The most distal part of the prolapse is closer than 1 cm to the hymen level.

Stage 3: The most distal part of the prolapse protrudes more than 1 cm from the hymen.

Stage 4: All or almost all of the vagina protrudes.

The evaluation of this classification is based on the “Pelvic Organ Prolapse Quantification system (POP-Q).”^[6]^

A rectocele consists of the anterior wall of the rectum protruding through a weakened recto-vaginal septum, creating a swelling in the posterior vaginal wall. The incidence of rectocele is unknown, but asymptomatic posterior compartment prolapse has been observed in approximately 40% of women who have given birth.^[7]^

Vaginal Birth is a process consisting of 4 phases. The first stage is characterized by painful uterine contractions and cervical dilation progressing to reach full dilation of the cervix. The second stage covers the period from full dilation of the cervix to the birth of the baby. The third stage is the part from the birth of the baby to the expulsion of the placenta and membranes. The fourth stage includes the first 4 hours after the placenta is expelled.^[8]^ The duration of the second stage of labor is stated as 2 hours in nulliparas and 1 hour in multiparas. In cases where the epidural is applied, adding one more hour to these periods is considered appropriate. If these periods are exceeded, interventional delivery is recommended with the diagnosis of a prolonged second stage.^[9]^ Long labor leads to many maternal and neonatal complications. Recently, the accuracy of the optimal limits for the duration of the first and second phases and the interventions used to accelerate the second phase have been discussed. Therefore, in this article, we have touched on one of the factors that may prolong the duration of the first and second stages of labor.

It starts in the first stage of labor when the first contractions begin and ends with full cervical dilation of up to 10 cm. The first stage of labor is further divided into 2 stages defined by the degree of cervical dilation. The latent phase is usually defined as 0 to 6 cm, while the active phase begins from 6 cm to full cervical dilation. The latent phase is usually significantly longer than that observed in the active phase and is less predictable in terms of the rate of cervical change. A normal latent phase can last up to 20 and 14 hours in nulliparous and multiparous women, respectively, without being considered prolonged. The use of sedation can prolong the latent phase of labor. The cervix varies more rapidly and predictably during the active phase until it reaches 10 cm and cervical dilation and effacement are finished. With faster cervical dilation, active action usually begins with a dilation of about 6 cm. During the active phase, the cervix typically dilates at a rate of 1.2 to 1.5 cm per hour. Multiparous women or women with a history of prior vaginal delivery tend to have more rapid cervical dilation. The absence of cervical change for more than 4 hours in the presence of enough contractions or more than 6 hours with insufficient contractions is considered as cessation of labor and may require clinical intervention.^[10]^

Prolongation of the latent phase;

- No transition to active phase for > 14 hours in multiparas and > 20 hours in nulliparas.

Prolongation of the active phase;

- Nullipara dilation progressing <1.2 cm/hour or progressing to <1 cm/hour.

Although there is no consensus, it has been mentioned in the literature that there is an opening rate of <1.5 cm/hour or a descent rate of <2 cm/hour in multipara.^[11]^

The second stage of labor includes the period until the completion of cervical dilatation and the delivery of the baby.^[12]^ The diagnosis of prolonged second stage in labor is accepted as the duration of the second stage of labor exceeding 3 hours in nulliparas and 2 hours in multiparas.^[12]^ Factors affecting the duration of the second stage of labor; Factors such as the first delivery after the age of 30, continuous electronic fetal monitoring and the use of narcotic analgesics in multiparous women are reported to be effective in prolonging the second stage of labor. It is also stated that the duration of the second phase of labor may be excessively prolonged due to epidural analgesia, sedation, and maternal fatigue.^[13]^

Prolongation of the stages of labor causes numerous maternal and neonatal complications.^[14]^ When we consider cystocele and rectocele as mechanical obstacles in the delivery path, they can extend the delivery time and lead to complications. There have been numerous studies in the literature regarding the relationship between vaginal delivery, cystocele, and rectocele. However, there are no studies investigating the maternal and neonatal outcomes of cystocele and rectocele during delivery. We aim to investigate the effects of cystocele and rectocele on vaginal delivery stages, as well as maternal and neonatal outcomes.

## 2. Methods

### 2.1. Study design

This prospective study was carried out at the Tertiary Care Gynecology and Obstetrics Clinic in Van. Hospital data from November 1, 2022, to February 28, 2023, were prospectively collected. The study protocol was approved by the Van Training and Research Hospital Ethics Committee. Written informed consent was obtained from all patients.

### 2.2. Participants

A total of 672 pregnant women between the ages of 18 to 40, who had a normal vaginal delivery and were at ≥ 37 weeks of pregnancy in our clinic were included in the study. No sample was selected in the study, all patients who met the acceptance criteria between the specified dates were included. Staging evaluation was performed according to the POP Quantification system.^[6]^

### 2.3. Variables

Patients who were hospitalized for reasons such as membrane ruptures that may affect the duration of birth, who had a cesarean delivery for various reasons (fetal distress, cephalo-pelvic disproportion, previous cesarean section), who had chronic diseases (such as hypertension and gestational diabetes, breech presentation and fetal macrosomia) were excluded from the study. Nulliparous patients were excluded due to longer labor stages and purer observation. The patients did not use any medications for medical treatment. Patients’ age, Body Mass Index, gravida, parity, prenatal and postnatal hemoglobin concentrations, cervical dilation time, cystocele stage, presence of rectocele, laceration, presence of chorioamnionitis, 1st and 5th minute Apgar scores, birth weight and neonatal intensive care unit need data were collected.

### 2.4. Statistical analysis

Statistical analysis was performed with the licensed SPSS 22.0 program. Categorical measurements are summarized as numbers and percentages, and continuous measurements are summarized as mean and standard deviation. The suitability of the data for normal distribution was evaluated with the Shapiro–Wilk test. In comparing continuous measurements between groups, One-Way ANOVA was used for more than 2 variables for parameters with normal distribution, and Kruskal Wallis tests were used for parameters that did not show normal distribution. Tukey HSD test was used as a Post Hoc analysis to determine differences between more than 2 groups. Chi-square and/or Fisher Exact test were used to evaluate categorical variables. In all tests, the statistical significance level was determined as α = 0.05.

## 3. Results

A total of 672 pregnant women were included in the study, of which 348. 348 (51.8%) had no abnormalities, 78 (11.6%) had rectocele only, 112 (16.7%) had cystocele only, and 134 (19.9) had both cystocele and rectocele The mean age of the pregnant women was 26.89 ± 5.94. All of the pregnant women gave birth vaginally.

The distribution of cystocele groups according to maternal characteristics is presented in Table 1. There was a significant differentiation between the groups in terms of parity, gravida, hemoglobin, cervical effacement 0 to 6 cm and 6-full patency time. In terms of gravida and parity, grade 0 is similar to grade 1, and grade 2 is similar to grade 3, with a significant differentiation between these groups. There was no differentiation between the groups in terms of fetal weight and age. In terms of hemoglobin, stages 0, 1, and 3 are similar but different from stage 2. There was no differentiation between the groups for the period from full cervical dilation to delivery. The reason why there is no differentiation between them while waiting for it to prolong due to prolapse may be the experience of the patients.

**Table 1:** Distribution of cystocele groups according to maternal characteristics

Maternal characteristics	Cystocele stage Mean (standard deviation)				
	Stage 0 (n:426)	Stage 1 (n:108)	Stage 2 (n:94)	Stage 3 (n:44)	*P* *
Age	26.30 (6.10)	26.422 (5.81)	28.18 (5.29)	29.30 (5.78)	.18
Parity	1.61 (1.38)^a^	1.59 (1.18)^a^	2.13 (0.94)^b^	2.14 (0.63)^b^	**<.001**
Gravida	2.77 (1.47)^a^	2.63 (1.06)^a^	3.33 (1.02)^b^	3.36 (0.57)^b^	**<.001**
Hemoglobin	13.02 (1.45)^a^	13.0 (0.98)^a^	12.18 (1.57)^b^	13.0 (1.23)^a^	**<.001**
Fetal weight	3175.60 (486.423)	3184.11 (323.799)	3183.72 (337.456)	3197.00 (305.668)	**<.08**
Cervical dilation 0–6 cm time	75.82 (30.41)^a^	73.20 (20.30)^a^	68.87 (18.44)^b^	86.81 (20.98)^c^	**<.01**
Cervical dilation 6–10 cm time	22.24 (11.22)^a^	22.25 (10.42)^a^	24.91 (9.75)^b^	30.95 (5.93)^c^	**<.001**
Full cervical dilation-born time	20.66 (15.86)	19.57 (14.48)	18.67 (13.49)	20.77 (14.97)	.43

* One-way ANOVA.

Normally distributed continuous variables are presented as mean ± SD. a, b, c shows the differences between the groups.

Table 2 shows the distribution of cervical erasure times according to cystocele grades in patients with rectocele and cystocele coexistence. There is a significant differentiation between the groups compared to those whose cervical effacement time is between 0 and 6 cm. Cervical effacement time is the highest in stage 3. There is a differentiation between the groups according to the cervical erasure time from 6 cm to full dilation. Cervical erasure time is maximum in stage 3. There is a differentiation between the groups according to the period of cervical effacement from fully open to delivery. The time from fully open to delivery, that is, according to the second stage of labor, is the longest in stage 3 and is significant compared to other groups.

**Table 2:** Dilatation distribution according to cystocele degrees in rectocele and cystocele coexistence.

	Cystocele stage Mean (standard deviation)				
	Stage 0 (n:78)	Stage 1 (n:59)	Stage 2 (n:52)	Stage 3 (n:23)	*P* *
Cervical dilation 0–6 cm	56.49 (4.89)^a^	58.60 (2.27)^a^	62.29 (5.34)^b^	70.00 (0.00)^c^	**<.001**
Cervical dilation 6–10 cm	22.31 (9.02)^a^	20.10 (5.75)^a^	19.50 (5.54)^a^	29.00 (0.00)^b^	**<.029**
Full cervical dilation-born	19.35 (17.95)^a^	20.08 (15.69)^a^	19.09 (7.82)^a^	22.88 (18.98)^b^	**<.007**

* One-way ANOVA.

Normally distributed continuous variables are presented as mean ± SD. a, b, c shows the differences between the groups.

Group distributions according to maternal characteristics are presented in Table 3. There was a significant differentiation between the groups in terms of age, parity, gravida, and hemoglobin BMI. The mean age of Group 1, Group 2, and Group 4 is higher than Group 3. Group 2 and Group 3 were significantly higher than the other groups in terms of parity. Groups 2 and 3 are similar in terms of gravida, but more than Group 1 and Group 4. The mean of gravida was higher in Group 2. In terms of hemoglobin, Group 1, Group 2, and Group 3 are similar but different from Group 4. The mean hemoglobin is the lowest in Group 4. There was no differentiation between the groups in terms of birth weight.

**Table 3:** Distribution of groups according to maternal characteristics.

	Group 1 Normal (n:348) Mean (SD)	Group 2 Rectocele (n:78) Mean (SD)	Group3 Cystocele (n:112) Mean (SD)	Group 4 Rectocele + Cystocele (n:134) Mean (SD)	*P* *
Age (yr)	26.51 (6.26)^a^	26.29 (6.61)^a^	21.50 (4.70)^b^	28.03 (4.99)^a^	**<.001**
Parity	1.52 (0.94)^a^	2.80 (1.93)^b^	2.42 (1.73)^b^	1.86 (1.29)^c^	**<.001**
Gravida	2.63 (1.01)^a^	4.24 (1.85)^b^	3.42 (1.73)^b^	2.99 (1.30)^c^	**<.001**
Hemoglobin (g/dL)	13.02 (1.52)^a^	13.08 (0.93)^a^	13.00 (0.00)^a^	12.49 (1.45)^b^	**<.004**
Fetal weight (g)	3175.60 (486.423)	3180.53 (334.55)	3180.00 (449.12)	3184.43 (396.01)	.09
Cervical dilation 0–6 cm	52.51 (12.31)^a^	53.52 (25.46)^a^	55.02 (9.55)^a^	68.24 (5.66)^b^	**<.007**
Cervical dilation 6–10 cm	23.87 (11.91)^a^	23.74 (7.24)^a^	22.12 (7.11)^a^	29.15 (8.21)^b^	**<.007**
Full cervical dilation-born	18.38 (16.89)^a^	18.19 (13.41)^a^	19.66 (17.96)^a^	23.50 (16.66)^b^	.006
Body mass index (kg/m^2^) n(%)†					
25 and below	79 (22.7)	14 (17.9)	19 (17.0)	9 (6.7)	**<.001** **
Over 25	269 (77.3)	64 (82.1)	93 (83.0)	125 (93.3)	

* One-way ANOVA.

** Chi-square test values in bold represent statistically significant results.

† Column percentages are given.

Normally distributed continuous variables are presented as mean ± SD. a, b, c shows the differences between the groups.

SD = standard deviation.

There was a significant differentiation between the groups in terms of cervical erasure time (0–6 and 6–10 cm), and this period was the longest in Group 4. There was no differentiation between Group 1, Group 2, and Group 3 according to cervical erasure time. Group 4 with the longest cervical erasure period, that is, patients with cystocele and rectocele coexistence. Group 4 was significantly longer than the other groups in terms of the time from fully opening to delivery, that is, the duration of the second stage of labor.

There is a significant differentiation between the groups according to body mass index. The incidence of cystocele and rectocele is higher in those with a body mass index above 25.

The distribution of groups according to neonatal characteristics is summarized in Table 4. There was no significant differentiation between the groups according to intensive care unit, small gestational age, Macrosomia, and APGAR scores.

**Table 4:** Distribution of groups according to neonatal outcomes.

		Group 1 Normal (n:348)	Group 2 Rectocele (n:78)	Group3 Cystocele (n:112)	Group 4 Rectocele + cystocele (n:134)	*P* *
		n (%)	n (%)	n (%)	n (%)	
Neonatal intensive care unit (NICU)	No	96 (32.4)	69 (23.3)	11 (3.7)	120 (40.5)	.558
	Yes	17 (43.6)	7 (17.9)	1 (2.6)	14 (35.9)	
Small for gestational age (SGA)	No	106 (38.9)	70 (33.3)	11 (5.6)	130 (22.2)	.421
	Yes	7 (38.9)	6 (33.3)	1 (5.6)	4 (22.2)	
Macrosomia birth weight (g)	No	107 (33.9)	75 (23.7)	12 (3.8)	122 (38.6)	.102
	Yes	6 (31.6)	1 (5.3)	0 (0.0)	12 (63.2)	
APGAR score Mean (SD)	1 min	7.74 (0.56)	7.61 (0.71)	7.50 (0.79)	7.79 (0.49)	.107**
	5 min	9.07 (0.31)	8.94 (0.42)	8.91 (0.28)	9.06 (0.35)	.062

* Chi-square test. Row percentages are given.

** One-way ANOVA.

APGAR = appearance (skin color), pulse (heart rate), grimace (reflex irritability), activity (muscle tone), and respiration, NICU = neonatal intensive care unit.

Group distributions according to maternal complications are presented in Table 5. There was a differentiation between the groups in terms of maternal laceration and the development of chorioamnionitis. The development of chorioamnionitis and laceration is significantly higher in mothers with coexisting rectocele and cystocele. In the evaluation of the antepartum and postpartum hemoglobin differentiations of the mothers according to the groups, although the hemoglobin loss increases from the normal group to the rectocele + cystocele group, this increase is not significant.

**Table 5:** Distribution of groups according to maternal complications.

		Group 1 Normal (n:348)	Group 2 Rectocele (n:78)	Group3 Cystocele (n:112)	Group 4 Rectocele + cystocele (n:134)	*P* *
Laceration (%)	No	340 (54.0)	73 (11.6)	102 (16.2)	114 (18.2)	**<.001** **
	Yes	8 (18.7)	5 (11.6)	10 (23.2)	20 (46.5)	
Chorioamnionitis (%)	No	440 (68.8)	72 (11.2)	11 (1.7)	117 (18.3)	**<.001** **
	Yes	8 (25.0)	6 (18,8)	1 (3,1)	17 (53,1)	
Bleeding†		1.29 (0.79)	1.29 (0.71)	1.37 (0.76)	1.45 (0.76)	.46*

* One-way ANOVA.

** Chi-square test values in bold represent statistically significant results. Row percentages are given.

† Mean ± SD.

## 4. Discussion

The duration of active labor is affected by factors such as advanced maternal age, nulliparity, labor induction, early amniotomy, fetal macrosomia, advanced gestational week, epidural analgesia, long contraction intervals, and fetal head malposition.^[15,16]^ Prolongation of the active phase despite adequate driving force often suggests cephalopelvic disproportion in obstetrics. Cephalopelvic disproportion and non-progressive labor diagnoses are the most common indications for cesarean section.^[17]^ According to our knowledge, although there are very few studies on the effect of pelvic relaxation on active labor in the literature, Declare stated that soft tissue may cause birth dystocia.^[18,19]^ However, cystocele and rectocele are not considered among the parameters that affect active labor. We thought that the mechanical obstruction of the soft tissues to the fetus, especially in the first and second stages of labor, may prolong the birth process. It is stated in the literature that the prolongation of the delivery period does not lead to good maternal and neonatal outcomes. We aimed to predict maternal and neonatal complications that may occur as a result of this and to reduce maternal and neonatal morbidity by predicting them.

As far as we know, very few studies have been done on the first stage of labor and the reasons for prolonging this stage have not been investigated in the literature. The study covers both the first stage and the second stage.

In the guide published by the World Health Organization in 2018 regarding the second stage of birth, it is stated that this period at birth may be different for each woman, and it will generally be completed within 3 hours in nulliparous patients and within 2 hours in multiparas.^[12]^ In the Obstetric Care Opinion (Obstetric Care Consensus No. 1) published in 2014, American College of Obstetrics and Gynecology (ACOG) similarly recommended waiting 3 hours in nulliparous patients and 2 hours in multiparas for the onset of contractions in the second stage of labor in cases where no maternal and fetal problems are detected.^[9,20]^ The reason why we did not find any patients who exceeded this period in our study is that we took these patients for cesarean section after 2 hours and excluded these patients from the study.

The World Health Organization 2018 guide emphasizes that a decision to hasten the second stage of labor is dependent on the health status of the mother and fetus, as well as the progression of labor. It states that when the condition of the woman and the fetus is favorable, there is no need for intervention as long as there is evidence of progress in the descent of the fetal head. However, the guide suggests that when the second stage exceeds the defined optimal duration, the chance of spontaneous delivery within an acceptable time decreases and intervention should be made to accelerate labor.^[12]^

The first information about the duration and management of the second stage was presented in the study by Friedman (1954). In most studies on this subject, the average duration in multiparous patients ranged between 12 and 20 minutes.^[21]^ In our study, the mean duration of the first and second stages of our patients was 39.32 ± 32 minutes. However, it was observed that it was significantly longer in the cystocele and rectocele groups compared to the other groups. We would expect only rectocele and cystocele to be longer due to soft tissue dystocia, but there was no differentiation between the 2 groups. The reason for the lack of differentiation compared to the normal group may be due to the high parity of the patients and their previous birth experiences.

There have been many studies on the prolongation of labor in the literature, and it has been suggested that prolongation of labor can lead to various maternal and neonatal complications. Many investigations have been focused on these complications, and most of them describe complications with the prolongation of the delivery period. In the Obstetric Care Opinion (Obstetric Care Consensus No. 1) published in 2014, ACOG indicated that there is no information on the effect of operative vaginal delivery practices, which are among the practices in reducing cesarean rates, on delivery times. ACOG suggested that these practices should be applied in the second stage of labor, in the presence of an experienced and well-trained physician^.[20]^ Additionally, intervented vaginal delivery used to safely induce or accelerate labor in the presence of 2 maternal and fetal indications; In cases of maternal exhaustion and inability to push effectively, or for medical indications (such as when maternal straining is contraindicated in the second stage of labor due to maternal cardiac problems), prolongation of the second stage of labor, cessation of fetal head progression and rotation, and unreliable fetal heartbeats in the second stage of labor, ACOG stated in 2015 that it should be implemented.^[22]^ Again, in NICE 2014 guideline, it is recommended that if the optimal period is exceeded, the woman should be directed to operative vaginal delivery.^[9]^ Another study, by Matta 2018, determined that prolonged labor was associated with operative delivery and maternal morbidity. Considering the evidence levels, the probability of performing a vaginal delivery with forceps is higher than with a vacuum application. However, with forceps application, 3rd to 4th degree perineal tears occur more frequently. On the other hand, it was determined that the probability of developing cephalohematoma increased as the time increased in vacuum applications. In addition, the sequential use of vacuum and forceps has been related to increased neonatal complications. Routine application of vacuum and forceps is not recommended.^[23]^ It has been stated in the literature that the information about the time period does not lead to better outcomes for women or their babies, rather than an unnecessary increase in the rate of cesarean section and interventions in vaginal deliveries.^[20,24]^

In another study, stage 3 cystocele can prolong the active labor period of labor in grand multiparous pregnant with pelvic relaxation. It has been argued that this effect of pelvic relaxation on the active phase may be an important factor in the decision to perform a cesarean section, considering the indication of birth dystocia.^[19]^

In a study by Laughon et al in 2014, they found that a prolonged second stage was associated with chorioamnionitis and third or 4th-degree lacerations.^[14]^ In our study, the risk of chorioamnionitis increased especially in patients with cystocele and rectocele. These patients were composed of patients with extended delivery periods. It is much more important that we closely monitor these patients.

In another study on prolonged labor, the relationship between delivery time and postpartum anemia was investigated; prolongation of labor was found to have a higher risk in terms of postpartum anemia.^[25]^ In our study, no significant differentiation was observed to be associated with a higher risk of postpartum anemia. The reason for this is that long labor is not the only risk factor for bleeding, and many risk factors may have affected it.

We excluded these patients from the study because of the high number of malpractice cases in our country, the low number of doctors with experience in operative vaginal delivery in our center, and the cesarean section of patients with prolonged delivery because of the thought that prolonged delivery would increase maternal and neonatal morbidity. Since we excluded these patients from the study, further maternal and neonatal morbidity was not included in our study. More research needs to be conducted on this subject. To reduce these complications, operative delivery is performed less and c/s orientation is increasing.

In ACOG 2019, it was mentioned that the person following in the second stage of labor should identify the signs and symptoms in the second stage of labor and provide continuous care, observation and support.^[26]^ Although reducing cesarean rates is an important goal, obstetric and neonatal complications in mothers and newborns should be considered.^[27]^

It is known that the duration of spontaneous delivery varies from 1 woman to another in women with good perinatal outcomes. In addition, some women may have a longer-than-expected vaginal delivery without adverse perinatal outcomes. It is recommended that these current optimal limits, which are currently used in clinical practice and used for prolonged second-stage diagnosis requiring obstetric intervention, be questioned. A definite consensus on the diagnosis of the prolonged second stage has not been reached yet. Although there are differentiations in the diagnosis of the prolonged second stage in the studies, it should not be ignored that the prolongation of the second stage of labor may cause maternal complications. Particularly, the reasons for prolonging the delivery should be reviewed. Although every cause does not produce the same results, possible complications in patients with cystocele and rectocele should be considered in advance, and the patient should be followed up and prepared for possible complications.

Our study has some limitations. These are inadequate operative delivery, different midwives and doctors who follow births in our center, not every doctor follow-up in the same way, patients who underwent cesarean section after birth and nulliparous patients were not included in the study. More research is needed on this topic.

## 5. Conclusion

The delivery time of pregnant women with cystocele and rectocele was found to be significantly longer than the control group in general, and our study has some limitations. Since these pregnant women did not have any additional risk factors, the prolongation of the active labor period was attributed to POP, specifically soft tissue dystocia. The duration of delivery in patients with cystocele and rectocele coexistence was found to be significantly longer compared to other patients. In the literature, it has been mentioned that the reasons for prolonging the delivery lead to unfavorable maternal and neonatal outcomes. In our study, maternal complications increased as well. Although we think that cystocele and rectocele are mechanical factors during delivery, due to the high parity and experience of these patients, the duration of delivery did not increase with the cystocele stage. However, patients with cystocele and rectocele coexistence should be more closely monitored due to possible complications during delivery, and we think that this criterion should be kept in mind when evaluating for cesarean section indication. Due to the low rate of operative delivery and the scarcity of experienced personnel in our hospital, operative vaginal delivery was not performed for the patients. We think that there is no difference in neonatal outcomes because we decided on cesarean section for these patients with the diagnosis of stopped labor. There are not enough studies on this subject and more detailed studies are needed on this subject.

## Author contributions

**Conceptualization:** Yusuf Başkiran, Kazim Uçkan, İzzet Çeleğen, Fatma Başak Tanoğlu.

**Data curation:** Yusuf Başkiran, Kazim Uçkan, İzzet Çeleğen, Fatma Başak Tanoğlu.

**Formal analysis:** Yusuf Başkiran, Kazim Uçkan, İzzet Çeleğen, Fatma Başak Tanoğlu.

**Funding acquisition:** Yusuf Başkiran, Kazim Uçkan, İzzet Çeleğen, Fatma Başak Tanoğlu.

**Investigation:** Yusuf Başkiran, Kazim Uçkan, İzzet Çeleğen, Fatma Başak Tanoğlu.

**Methodology:** Yusuf Başkiran, Kazim Uçkan, İzzet Çeleğen, Fatma Başak Tanoğlu.

**Project administration:** Yusuf Başkiran, Kazim Uçkan, İzzet Çeleğen, Fatma Başak Tanoğlu.

**Resources:** Yusuf Başkiran, Kazim Uçkan, İzzet Çeleğen, Fatma Başak Tanoğlu.

**Software:** Yusuf Başkiran, Kazim Uçkan, İzzet Çeleğen, Fatma Başak Tanoğlu.

**Supervision:** Yusuf Başkiran, Kazim Uçkan, İzzet Çeleğen, Fatma Başak Tanoğlu.

**Validation:** Yusuf Başkiran, Kazim Uçkan, İzzet Çeleğen, Fatma Başak Tanoğlu.

**Visualization:** Yusuf Başkiran, Kazim Uçkan, İzzet Çeleğen, Fatma Başak Tanoğlu.

**Writing – original draft:** Yusuf Başkiran, Kazim Uçkan, İzzet Çeleğen, Fatma Başak Tanoğlu.

**Writing – review & editing:** Yusuf Başkiran, Kazim Uçkan, İzzet Çeleğen, Fatma Başak Tanoğlu.
